# Neuralgia—Its Pathology and Etiology

**Published:** 1881-05

**Authors:** S. S. Wilson

**Affiliations:** Xenia, Ohio.


AMERICAN JOURNAL

OF

### DENTAL SCIENCE.

Vol. XV. THIRD SERIES—MAY, 1881. No. 1.

### ARTICLE I.

### *Neuralgia—Its Pathology and Etiology.*

BY S. S. WILSON, M. D., XENIA, OHIO.

True neuralgias do not claim a place among the most frequent diseases of the human system, yet they are sufficiently common to be certain to occur with more or less frequency in the practice of every doctor, whether he be an M. D. or a D. D. S., and their importance is not to be measured by the frequency with which they occur, but rather by the extreme suffering which they inflict, and by the too often successful rebellion which they wage against our efforts to conquer them, or even mitigate the agony which they occasion. The pain which they inflict, and their rebelliousness to treatment, place them among the sources of greatest anxiety to the practitioner. No class of diseases is better calculated to teach the physician (and the dentist as well) the importance of his most strenuous efforts; none is more likely to occasion mortification on account of repeated failure, and none more likely to occasion the loss of professional credit.

This condition of things is largely due to the extreme timidity and vagueness with which our leading authors are in the habit of speaking of the pathology and etiology of this disease. It must be admitted that the pathology of neuralgia is not as clearly defined as that of some other diseases, yet, when taken in connection with its etiology, we can obtain sufficient light upon the nature of the disease to serve almost all practical purposes.

In this paper I shall endeavor to establish the following points, viz.: (*a*) That in the vast majority of cases, neuralgic pains are due to nerve-lesions of central origin. (*b*) That the essential seat of this lesion is the posterior root of the spinal nerve in which the pain is felt; and (*c*) that the essential condition of that nerve root is atrophy. One of the strongest arguments which we possess, in confirmation of the first of these points, is the undoubted hereditary nature of neuralgia. This character of the disease is a matter of great importance, a fact that the practitioner must readily admit, if he will only follow out the course of questioning and reasoning which it indicates. The minor position among diseases which is given to neuralgia, by the common people, is the source of much difficulty in obtaining a reliable family history on this point, farther back than one or two generations; but instances in which it can be traced for three, and even four generations, are of sufficient frequency to enable us to draw positive conclusions. In pursuing our examination we should not content ourselves with the history of those who have been actually neuralgic, but we should look farther and take in all those other serious neuroses, such as insanity, epilepsy, hypochondria, paralysis, chorea, etc., to all of which neuralgia is more or less nearly allied. Much difficulty is always encountered in conducting such an examination, but if it is faithfully carried out, it must convince any candid mind that neuralgia is notably a hereditary disease. The following striking example has fallen under the notice of the writer: Three generations of a family are now living, and from the oldest of these we have received what we have good reason to believe is a faithful history of the generation last deceased, which was of a marked neuralgic tendency. The grandfather, the oldest of the family now living, until sixty years of age, suffered from frequent and violent attacks of trigeminal neuralgia, and has always been nervous and extremely irritable. A brother of his also suffered with neuralgia, affecting the fifth nerve, and had one child imperfectly developed, both in body and in mind. This child, at death, was a lunatic, about four feet in height, with small body, but with enormously developed head. In another branch of the family, three sons are insane, one has always been melancholy, and one was born blind. Of the second generation, which compose a family of nine children, not a single one has escaped the neuralgic tendency, the fifth nerve being the usual seat of the difficulty. One of the number is decidedly hysterical, and another suffers frequently from spasmodic asthma. Of the grand-children, now numbering near twenty, four have been troubled with spasmodic croup; one became a stutterer at five years of age, and is still so at twelve years of age; and five suffered from trigeminal neuralgia. What nervous troubles may be developed hereafter, in the rising generation, cannot now be ascertained; but if the past is any indication of the future, the lives of these individuals will be loaded with much of evil of a neurotic inheritance. If more extended proof of the hereditary nature of neuralgia is desired, I would refer any who may be skeptical on this point to the history of neuralgic cases in their own practice; to the record of twenty-two cases given by Austin, derived from his private practice; and also to an analysis, by the same author, of eighty-three cases, hospital and private patients, whose blood relations had suffered from various of the neuroses. In addition, we frequently see two or more of the neuroses alternating with each other, or the one following the other as the result or climax; as when neuralgia follows mental agitation, or where insanity follows neuralgia, or, as in Dr. Mandsley's patient, neuralgia and insanity alternating with each other, the one vanishing during the prevalence of the other. In such cases the lesion is evidently central, and the alternation is due merely to the transition of the affection from one centre to another.

The pains of locomotor ataxy deserve a prominent place in the treatment of this subject. In this disease we have an example of the heredity of nervous troubles. Not only do we find a tendency to it transmitted from generation to generation, but we will find it existing in two or more members of the same family. So marked is this disposition, that when we meet with those shifting, lancinating pains so characteristic of this disease, it becomes us to make diligent inquiry as to the existence of the disease among the ancestors of our patient. In this disease also, there is no doubt as to the actual seat of the lesion, nor as to the character of the lesion, the posterior columns of the cord, together with the posterior nerve roots, being the parts chiefly affected, and the condition of these parts being that of atrophy.

Important testimony bearing upon this subject is to be derived from the history of cases of spinal paralysis. I have in mind two cases of paraplegia, in which the most violent neuralgic pains occurred in the nerve trunks given off from the cord below the point of injury. In these cases the position and nature of the lesion could hardly be mistaken.

Chronic alcoholism, by the production of arterial and capillary degeneration, cuts off to a certain extent the nutrition supply to the cord and nerve roots, and thus gives rise to pains very much resembling those of neuralgia.

The development of the genito-urinary apparatus probably has more to do with the causation of neuralgia than most physicans suppose. It is a very noticeable fact that nearly all cases of epilepsy, migraim and clavus make their appearance during the period of sexual development. During this period, special demands are being made upon the system to complete the development of these special organs; and, unfortunately, this is a time of life when ambitious children, and the mistaken ideas of ambitious parents, and the superlative foolishness of teachers and boards of education, demand that the mental powers of the child shall be taxed to the utmost. Under such circumstances it is but natural to suppose, what is really the case, that portions of the nervous system not directly concerned in the development of either the mental or sexual organs must suffer from a lack of nutrition. The mudella oblongata is the part of the nervous system which must specially suffer at this time, and as a result of this, we have disorderly nervous action, producing explosive manifestations (epilepsy) in the motor apparatus, and pain (neuralgia) in the sensory.

In closing this paper we will make a hasty notice of some of the external influences which take part in the causation of neuralgia, and while speaking of them as external causes, we are convinced that in the majority of cases they are only exciting causes, their action being effective usually only in cases where the centers of the nerves exposed to their influence already possess a neuralgic disposition.

In the continued application of extreme cold, or of cold and moisture to the naked skin, we have a cause of neuralgia which is frequently effective; but while we say that it is frequently effective, we must add, that neuralgia is by no means a common sequence of such exposure; for when we consider the vast number of persons who are constantly exposed, we must at once conclude that if this were a very powerful factor in the production of neuralgia, we should, during the winter months, be over-run with such patients. The same thing is true of injuries to nerve trunks, and in these two examples of peripheral causes of neuralgia, we have an argument in favor of our first proposition, that the essential seat of the lesion in neuralgia is central; and the nerve root is lowered in vitality. Nature has performed experiments in abundance for the proof of this. The greatest amount of pain is felt not at the time of the application of the cold, nor at the time of the receipt of the injury, but during the period when the nerve is returning to its normal condition, the period when the nerve-centre is being exhausted of its reserve of nerve force, in re-establishing full functional activity in the parts in which this has been abolished by the action of cold or violence. The pressure of tumors, and especially of aneurismal tumors, upon the posterior roots of nerves or upon their ganglia, gives rise to severe neuralgic pains, and post-mortem examinations of these cases reveal the fact that the parts subject to pressure are in a state of atrophy, more or less advanced, according to the amount of pressure, and it teaches this important fact in this connection, viz: that the most acute neuralgic pain may be felt in the peripheral termination of a nerve, the central end of which is almost completely destroyed.

Other sources of peripheral causes are found in errors in the performance of the functions of the digestive, and of the genito-urinary organs ; in errors in the nutrition of the teeth, and in the performance of the functions of the eye ; but on these, time and space will not allow us to dwell, and, content with their mention, we close our paper, with the hope that it may be of as much benefit to some reader as its preparation has been to the writer.—*Ohio Journal of Dental Science.*

